# THIS TOO SHALL PASS: WEATHERING THE STORM AS OLDER FEMALE FAMILY CAREGIVERS FOR THOSE WITH AD/ADRD DURING COVID-19

**DOI:** 10.1093/geroni/igac059.348

**Published:** 2022-12-20

**Authors:** Candace Harrington, Dean Witt

**Affiliations:** University of Louisville, Louisville, Kentucky, United States; University of Louisville, Harrodsburg, Kentucky, United States

## Abstract

**Background:**

Older female caregivers of persons with AD/ADRD are under-represented, under-reported, and understudied. Purpose: This qualitative study aimed to understand how COVID-19 affects older female caregivers' lived experience, ongoing capacity, and willingness to provide care for their loved one(s) with AD/ADRD. Specific Aims: Aim 1: Explicate older female caregivers' lived experience in the context of caring for family members with AD/ADRD during COVID-19. Aim 2: Elucidate how COVID-19 affected older female caregivers' relationships with their family members with AD/ADRD. Method: 172 units of meaning were extracted from 327 pages of transcripts and 972 minutes of interviews with urban (n = 10) and rural caregivers (n = 10). Thematic analysis was then conducted.

**Results:**

Respondents, recruited with purposive and snowball sampling, saw hastened AD/ADRD progression in their family member(s) attributed to social isolation. For many, long-term placement was not an option for financial reasons. Respondents providing full-time caregiving depended heavily on their care recipients' financial resources for basic living expenses, reporting placement in long-term care would leave them at risk of homelessness. Black caregivers expressed an "unspoken" cultural taboo about placement. For all, caregiver disability was the only impetus for placement. Respondents in rural communities more often reported faith-based gratefulness, moments of joy, relational harmony resulting from "being stuck together", and less availability of resources allowing virtual support. Urban caregivers reported more social isolation, less awareness of resources, more intentional family member distancing, and higher pandemic-related distress. Implications: These findings have broad economic, social, policy, research, and practice implications.

